# The role of histone methyltransferases in neurocognitive disorders associated with brain size abnormalities

**DOI:** 10.3389/fnins.2023.989109

**Published:** 2023-02-10

**Authors:** Foster D. Ritchie, Sofia B. Lizarraga

**Affiliations:** Department of Biological Sciences and Center for Childhood Neurotherapeutics, University of South Carolina, Columbia, SC, United States

**Keywords:** autism, histone methyltransferase, brain size, neurodevelopment, chromatin, microcephaly, macrocephaly

## Abstract

Brain size is controlled by several factors during neuronal development, including neural progenitor proliferation, neuronal arborization, gliogenesis, cell death, and synaptogenesis. Multiple neurodevelopmental disorders have co-morbid brain size abnormalities, such as microcephaly and macrocephaly. Mutations in histone methyltransferases that modify histone H3 on Lysine 36 and Lysine 4 (H3K36 and H3K4) have been identified in neurodevelopmental disorders involving both microcephaly and macrocephaly. H3K36 and H3K4 methylation are both associated with transcriptional activation and are proposed to sterically hinder the repressive activity of the Polycomb Repressor Complex 2 (PRC2). During neuronal development, tri-methylation of H3K27 (H3K27me3) by PRC2 leads to genome wide transcriptional repression of genes that regulate cell fate transitions and neuronal arborization. Here we provide a review of neurodevelopmental processes and disorders associated with H3K36 and H3K4 histone methyltransferases, with emphasis on processes that contribute to brain size abnormalities. Additionally, we discuss how the counteracting activities of H3K36 and H3K4 modifying enzymes vs. PRC2 could contribute to brain size abnormalities which is an underexplored mechanism in relation to brain size control.

## 1. Introduction

Neurodevelopment is a complex process that depends on the precise regulation of gene transcription. In particular, the development of the cerebral cortex, the structure in the brain that gives us our highest cognitive functions, is reliant on the careful orchestration of multiple cellular processes including the proliferation of neuronal progenitors, as well as the migration, and differentiation of neuronal cells. Early in development a thin neuroepithelium constituted by neuroepithelial stem cells (NES) lines up the ventricles of the developing telencephalon. As development proceeds, NES give rise to Radial Glial Cells (RGCs) which are neuronal progenitor cells (NPCs) located in the ventricular zone (VZ) (Namba and Huttner, 2017). RGCs have an apical and a basal process that sense signals from the environment. In addition, the RGC basal processes also provide structural support for the migration of the newly born neurons (Noctor et al., 2002). In lyssencephalic mammals (e.g., mice), a second proliferative region is located away from the ventricle in the subventricular zone (SVZ). The NPC population at the SVZ is composed of both outer (or basal) Radial Glia Cells (oRGCs) and intermediate progenitor cells (IPCs) which lack an apical process (Namba and Huttner, 2017). In gyrated mammals (e.g., ferrets and primates) there is an expansion of the SVZ forming an inner (iSVZ) and an outer (oSVZ) SVZ (Lui et al., 2011). In particular, the oSVZ composed of both IPCs and oRGCs is proposed to have driven the rapid evolutionary expansion of the superficial layers of the cerebral cortex contributing to a larger brain to body size ratio in humans compared to other non-human primates (Lui et al., 2011; Nowakowski et al., 2016).

Changes in the mode of NPC cell division are proposed to contribute to the diversity of the daughter cell population. Before the onset of neurogenesis, NES and RGC cells divide symmetrically to generate two identical daughter cells (Brand and Rakic, 1979). This mode of cell division occurs with the division plane perpendicular to the neuroepithelial ventricular surface. After the start of neurogenesis at the VZ, RGCs undergo asymmetric or symmetric cell division. In RGCs, a parallel or oblique plane of division with respect to the neuroepithelium ventricular surface is proposed to result in the asymmetric inheritance of the basal process and cell fate determining factors giving rise to two distinct daughter cells–an NPC and a neuron for example (Chenn and McConnell, 1995; Huttner and Brand, 1997; Haubensak et al., 2004). In contrast, oRGCs undergo proliferative symmetric cell divisions to expand the NPC population, or non-proliferative symmetric terminal divisions to give rise to two post-mitotic neurons (Shitamukai et al., 2011).

After birth newly born neurons migrate away from the VZ to their final location into the nascent cortical plate along the RGC basal processes. The earliest born neurons will form the pre-plate that later on will be split into the marginal zone and sub-plate by the first wave of migrating neurons. Subsequent waves of newborn neurons will form the transcriptionally and functionally distinct layers in the neocortex (Molnar et al., 2019a,b). Early in neurogenesis the earlier born projection neurons populate the deeper layers of the cerebral cortex, while during mid-neurogenesis the later born neurons start to populate the more superficial layers of the developing cortex (Rakic, 1972, 2009; Clowry et al., 2010). As neurogenesis ends, there is a switch from a neurogenic to a gliogenic fate, for which RGC competence changes to generate glial cells (astrocytes and oligodendrocytes) at late embryonic or perinatal stages (Ohtsuka and Kageyama, 2019).

Improper regulation of NPC division during cortical development has been proposed as a major mechanism underlying defects in brain size. Primary microcephaly has been associated with mutations in genes that modulate centrosome function and mitotic spindle formation, which are essential for proper cell division (Cox et al., 2006; Jean et al., 2020). Alternatively, postnatal microcephaly, or the failure of the brain to grow after birth (Seltzer and Paciorkowski, 2014), has been associated (albeit not exclusively) with mutations in transcriptional and chromatin regulators that modulate the expression of genes important to the development of the forebrain and hindbrain (Seltzer and Paciorkowski, 2014). In contrast, an increased head size, or macrocephaly, has also been associated with mutations in developmentally important genes that regulate neurogenesis and gliogenesis (Chenn and Walsh, 2002; Geisert et al., 2002; McCaffery and Deutsch, 2005). Alterations in brain size could ultimately alter the cytoarchitecture of the developing brain and impair the development of the neuronal circuitry. Not surprisingly, microcephaly has been associated with disorders of neuronal connectivity including autism spectrum disorders (ASD), and intellectual disability (ID), while macrocephaly has been primarily correlated with ASD (Fombonne et al., 1999; Miles et al., 2000).

Proper development of the brain architecture and wiring requires the careful regulation of multiple gene programs that may need to seamlessly transition between transcriptional activation or repression states in a cell type specific manner at distinct developmental timepoints. Epigenetic mechanisms such as DNA methylation, histone post-translational modifications, and chromatin remodeling are essential for these transitions. Histone methylation is a type of post-translational histone modification that is highly dynamic and essential to the regulation of transcriptional activation and repression. Work from multiple experimental systems has demonstrated that during development histone methylation is essential for the establishment of different cell lineages (Jambhekar et al., 2019). For instance, the evolutionarily conserved Polycomb Group of Proteins Complex (PcG) is a major regulator of histone methylation that is essential for the switching between neurogenic to gliogenic fates in the developing cerebral cortex (Pereira et al., 2010). Therefore, the precise regulation of a wide array of distinct histone methylation marks modulates transcriptional programs in a spatial and temporal manner to ensure normal brain development (Jambhekar et al., 2019).

**FIGURE 1:**
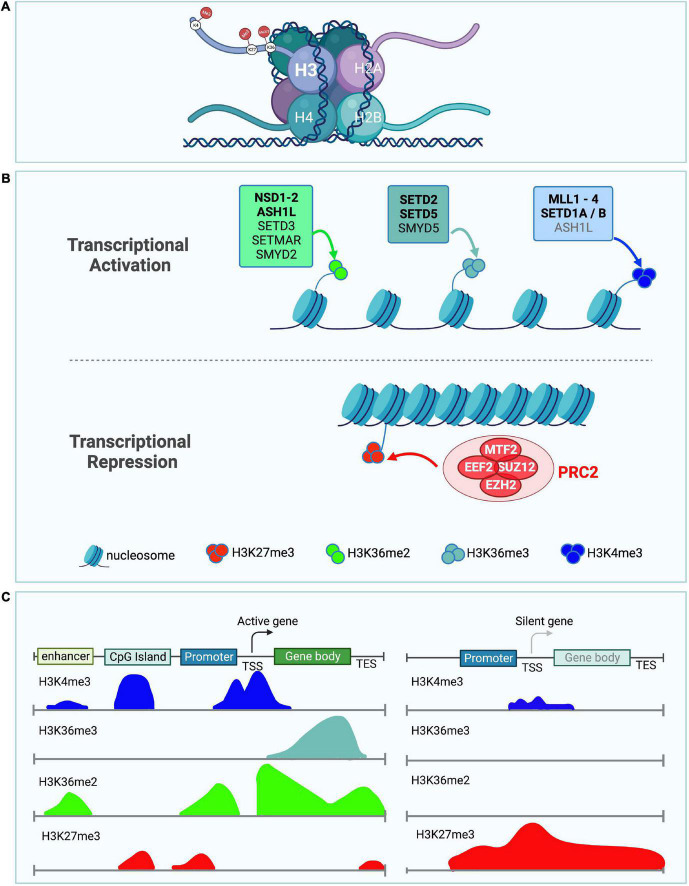
Role of H3K36me2/3, H3K4me3, and H3K27me3 in transcriptional activation and repression. **(A)** Depiction of a nucleosome showing the location of the different lysine histone modifications in the tail of histone H3. **(B)** Schematic showing histone methyltransferases and their corresponding histone marks associated with transcriptional activation (top panel) and repression (bottom panel). H3K36me2 (light green), H3K36me3 (light blue), and H3K4me3 (dark blue) histone marks are associated with transcriptional activation. Histone methyltransferases associated with deposition of either H3K36me2, H3K36me3, or H3K4me3 are shown. ASH1L is in gray for H3K4me3 as it might indirectly affect the levels of this histone mark. Bold enzyme names are associated with neurodevelopmental disorders or have neuronal phenotypes. H3K27me3 (red) histone mark is associated with transcriptional repression. The PRC2 complex catalyzes H3K27 methylation, its individual core subunits are shown in red. **(C)** Diagram illustrates the distribution of the different histone marks in different genomic locations including the enhancer, CpG island, promoter, transcription start site (TSS), gene body, and transcription end site (TES). Left panel depicts the histone modification distribution in a transcriptionally active gene. Right panel shows histone modifications in a transcriptionally silent gene (Lim et al., 2010).

**FIGURE 2:**
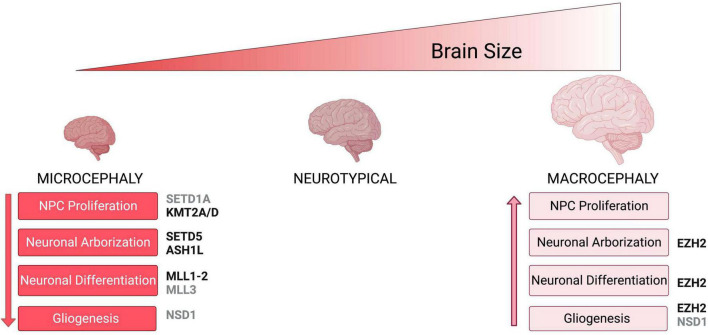
Neuronal phenotypes associated with brain size abnormalities. Schematic shows brain size abnormalities and potential cellular defects associated with differences in brain size. Microcephaly mechanism are shown in red while macrocephaly mechanisms are shown in light pink. Histone methyltransferases and their corresponding neuronal phenotypes that have been associated with either microcephaly or macrocephaly are shown in black, while enzymes for which there is no conclusive evidence on specific phenotypes are shown in gray.

In particular, histone methyltransferases that modify Histone H3 on Lysines 4, 36, and 27 are emerging as major regulators of neuronal structure and function (Figures 1A–C). Tri-methylation of Histone H3 on Lysine 4 (H3K4me3) and di or tri-methylation of Histone H3 on Lysine 36 (H3K36me2 and H3K36me3) have been associated with transcriptional activation (Bannister et al., 2005; Barski et al., 2007; Wagner and Carpenter, 2012; Zhu et al., 2013; Tables 1–4). In contrast to the activating effect on transcription of H3K4me3 and H3K36me2/3, tri-methylation of Histone H3 on Lysine 27 (H3K27me3) (Yu J. et al., 2019) by the Polycomb repressor complex 2 (PRC2) has been linked to transcriptional silencing. Histone modifications can either facilitate or hinder the recruitment or activation of other chromatin factors (Kim and Kim, 2012; Gates et al., 2017). Both H3K36me2/3 and H3K4me3 have been proposed to either directly or indirectly sterically hinder the deposition of H3K27me3 by the PcG which acts as a transcriptional repressor during development (Tie et al., 2014; Schuettengruber et al., 2017). While these mechanisms have been widely studied in cancer and general development, we focused on the evolutionary conserved counteracting activities of H3K36 (SETD2, SETD5, NSD1, NSD2, and ASH1L), and H3K4 (MLL1/KMT2A, MLL2/KMT2B, MLL3/KMT2C, MLL4/KMT2D, SETD1A, and SETD1B) modifiers vs. PcG (PRC2), because their antagonizing activities are understudied in the regulation of brain architecture, wiring and brain size control (Figures 1, 2 and Tables 1–4).

**TABLE 1:** Neurodevelopmental disorders associated with H3K36 histone methyltransferases.

Enzyme	Histone mark	Associated neurodevelopmental disorders	Brain size alterations	Brain size defects source
NSD1	H3K36me1/2	Sotos syndrome (ID, ASD)	Macrocephaly (79/85) and (16/16)	Faravelli, 2005; Muhsin et al., 2022
		Microduplication 5q35 syndrome	Microcephaly (29/39)	Quintero-Rivera et al., 2021
NSD2	H3K36me2	Wolf-Hirschhorn syndrome-DD, ID	Microcephaly (14/28) and (6/7)	Barrie et al., 2019; Zanoni et al., 2021
ASH1L	H3K36me1/2	ASD, ID, seizures	Microcephaly (1/1) and (1/7)	Okamoto et al., 2017; Faundes et al., 2018
			Macrocephaly (1/1)	Wang et al., 2016
		ADHD, motor, and speech delay	Normocephalic (1/1)	Shen et al., 2019
		Tourette Syndrome/ADHD	Not ascertained	Liu et al., 2020
SETD2	H3K36me3	Overgrowth Syndrome (ASD, ID)	Macrocephaly (10/14) and (11/13)	Marzin et al., 2019; Chen et al., 2021
		NDD, severe ID (p. Arg1740Trp)	Microcephaly (12/12)	Rabin et al., 2020
SETD5	H3K36me3	SETD5-associated KBG syndrome	Microcephaly (1/2)	Crippa et al., 2020
		3p25 Microdeletion Syndrome (ID, seizures)	Microcephaly (3/4) and (2/4)	Grozeva et al., 2014; Kuechler et al., 2015
		ASD/variable ID/seizures/motor and speech delays	Not ascertained	Fernandes et al., 2018
			Normocephalic (12/14)	Powis et al., 2018

This table shows H3K36 writers, that have corresponding neurodevelopmental syndromes. The corresponding histone modifications are shown. The brain size defects associated with mutations in the different genes are shown. References shown are related primarily to reports in which brain size differences were reported.

**TABLE 2:** Neuronal phenotypes associated with mutations in H3K36 histone methyltransferases.

Enzyme	Species	Experimental design	Phenotypes	References
NSD1	Mouse	shRNA KD in embryonic brain	Impaired neuronal migration	Almuriekhi et al., 2015
		Heterozygous KO	Normal cerebral cortical size and progenitor number, fewer SATB2 neurons in Retrospenial cortex, UV vocalization defects	Oishi et al., 2020
	Drosophila	Overexpression in glial cells	Increased apoptosis neuronal and glial cells; decreased head/brain size; learning defects	Kim et al., 2020
NSD2	Zebrafish	KD by morpholinos	Enlargement of endbrain/cerebral ventricle; Ventrally projecting neurons are truncated; Reduced numbers of motor neurons	Yamada-Okabe et al., 2010
		Homozygous KO	Smaller body length; Enlarged Diencephalic ventricle; and Reduced number of motor neurons	Yu et al., 2017
	Mouse	Homozygous KO	Decreased growth rate	Nimura et al., 2009
		Wolf-Hirschhorn deletion model (radiation induced)	Reduced cerebral cortex size, cerebellar foliage defects, learning deficits	Naf et al., 2001
ASH1L	Human	CRISPRi KD iPSC-derived neural progenitor cells	Reduced neural progenitor cell proliferation; and reduction in neurite outgrowth	Lalli et al., 2020
		shRNA KD in ESC-derived cortical neurons	Reduced neurite length and complexity; enlarged growth cone and cell bodies	Cheon et al., 2022
	Mouse	Nestin-CRE-KO	Growth delays; cortical layer disorganization, reduced sociability, and increased anxiety-like behaviors.	Gao et al., 2021a,b
		Haploinsufficiency model and EMX-CRE-KO	Neuronal hyperactivity; deficits in synapse pruning, propensity to seizures	Gao et al., 2022; Yan et al., 2022
SETD2	Mouse	Forebrain KO	Defects in neocortical arealization, cortico-thalamo-cortical circuits, social interaction, motor learning, and spatial memory	Xu et al., 2021
		*In utero* KD at E14	Defects in polarity and neuronal migration	Xie et al., 2021
SETD5	Mouse	shRNA KD	Increased proliferation of cortical progenitor cells	Sessa et al., 2019
		Heterozygous mutant	Decreased body size, glutamatergic synapse formation, neuronal activity, and deficits in social interaction	
		Haploinsufficiency model	Normal brain size and cortical lamination; delayed ultrasonic vocalization, impairments in cognitive tasks and behavioral inflexibility.	Deliu et al., 2018
		Haploinsufficiency	Decreased neurite length and complexity, thinner layer V (CTIP2), reduced firing rate in primary cultures	Moore et al., 2019

This table shows H3K36 writers described in Table 1. We show the corresponding species, experimental system, neural relevant phenotypes, and the respective literature citations.

**TABLE 3:** Neurodevelopmental disorders associated with H3K4 histone methyltransferases.

Enzyme	Histone mark	Associated neurodevelopmental disorders	Brain size alterations	Brain size defects source
SETD1A	H3K4me3	Novel SETD1A-Associated NDD (global DD, ID, behavioral problems)	Macrocephaly (3/13)	Kummeling et al., 2021
		Schizophrenia	Not reported	Singh et al., 2016
SETD1B	H3K4me3	SETD1B associated syndrome (ID, DD, ASD, seizures)	Microcephaly (2/36)	Weerts et al., 2021
			Macrocephaly (2/36)	
MLL1/KMT2A	H3K4me1/2/3	Weidemann–Steiner Syndrome (DD/ID, ASD, pre/post-natal growth deficits, seizures, corpus callosum defects)	Microcephaly (11/71)	Sheppard et al., 2021
			Microcephaly (2/6)	Chan et al., 2019
		Kabuki Syndrome (mild-moderate cognitive disability, postnatal growth deficits)	Microcephaly (2/2)	Sobreira et al., 2017
		Rubinstein–Taybi syndrome (ID, Speech delay, postnatal growth retardation)	Microcephaly (3/7)	Castiglioni et al., 2022
MLL2/KMT2B	H3K4me3	Early childhood onset Dystonia (motor, psychomotor defects, ID, ADHD, seizures)	Microcephaly (4/4)	Kawarai et al., 2018
			Microcephaly (2/19)	Meyer et al., 2017
		Non-dystonia syndrome	Microcephaly (17/44)	Cif et al., 2020
MLL3/KMT2C	H3K4me1/2/3	Kleefstra Syndrome (ID, seizures, behavioral problems)	Microcephaly (1/4)	Kleefstra et al., 2012
		ID/ASD related disorder (variable ID severity, ASD, language/motor delay, short stature)	Microcephaly (3/6)	Koemans et al., 2017
MLL4/KMT2D	H3K4me1/2	Kabuki Syndrome (ID, pre and post-natal short stature, seizures)	Microcephaly (7/26)	Lehman et al., 2017
			Microcephaly (11/37)	Usluer et al., 2022

This table shows H3K4 writers, that have corresponding neurodevelopmental syndromes. The corresponding histone modifications are shown. The brain size defects associated with mutations in the different genes are shown. References shown are related primarily to reports in which brain size differences were reported.

**TABLE 4:** Neuronal phenotypes associated with mutations in H3K4 histone methyltransferases.

Enzyme	System	Experimental design	Phenotype	References
SETD1A	Mouse	Frameshift mutation exon 7 heterozygous mutants and KD	Reduced sociability, severe hyperactivity, reduced synaptic function, and spine density	Nagahama et al., 2020
		LoF Heterozygous KO	Deficits in working memory, alterations in PFC synaptic function, reduced axonal branching and synaptic density	Mukai et al., 2019
	Human	iPSC-neurons Haploinsufficiency CRISPR-induced deletion of exon 7	Increased dendritic complexity, increased network activity, increased cAMP signaling	Wang et al., 2022
SETD1B	Mouse	CamKII-CRE-KO Postnatal deletion excitatory neurons	Severe learning impairment	Michurina et al., 2022
MLL1/KMT2A	Zebrafish	KD by morpholino and dominant negative OE	Reduced NPC proliferation, premature neuronal differentiation, reduced gliogenesis	Huang et al., 2015
	Mouse	cKO excitatory forebrain neurons	Impaired hippocampal memory formation	Kerimoglu et al., 2017
		hGFAP-CRE/MLL1 cKO NPCs SVZ, SGZ, cerebellar granule cells	Reduced size of cerebellar granule layer and hippocampal dentate gyrus. Impaired SVZ neurogenesis but not gliogenesis	Lim et al., 2009
		Heterozygous mutant	Decreased spine density in basal ganglia and increased aggressive behavior	Vallianatos et al., 2020
MLL2/KMT2B	Mouse	KO in ESCs	Delayed differentiation toward ectodermal lineage and increased apoptosis of ESCs	Lubitz et al., 2007
		KO in embryonic fibroblasts	Impaired trans differentiation of fibroblasts into neuronal cells	Barbagiovanni et al., 2018
		cKO postnatal forebrain excitatory neurons	Impaired hippocampus dependent memory formation	Kerimoglu et al., 2013
MLL3/KMT2C	Rat	siRNA KD in cortical neurons	Hyperactive neuronal networks during development; Reduced inhibitory and excitatory synaptic puncta	Frega et al., 2020
	Drosophila	RNAi in the adult nervous system	Reduced short term memory	Koemans et al., 2017
MLL4/KMT2D	Human	iPSC-derived NPCs from a Kabuki syndrome patient	Reduced NPC proliferation	Carosso et al., 2019
	Mouse	KMT2D/β-geo-truncated protein lacking SET domain	Hippocampal memory dysfunction, reduced neurogenesis of granule cell layer	Bjornsson et al., 2014

This table shows H3K4 writers described in Table 3. The corresponding species, experimental system, neural relevant phenotypes, and the respective literature citations are shown.

## 2. H3K36 methylation and neurodevelopment

Methylation of Lysine 36 on Histone H3 (H3K36) has important biological implications due to its involvement in the regulation of transcriptional activation, splicing, and the control of spurious transcription (Wang et al., 2007; Pradeepa et al., 2012; Huang and Zhu, 2018). H3K36 methylation occurs in three different forms: mono-methylation (H3K36me), di-methylation (H3K36me2), and tri-methylation (H3K36me3). H3K36me is widely considered an intermediate step, but a function for H3K36me in the repair of double-strand breaks has been reported (Jha and Strahl, 2014). Both H3K36me2 and H3K36me3 are mainly contained within lightly packed regions of chromatin, indicating their role in transcriptional activation (Bannister et al., 2005; Wagner and Carpenter, 2012; Figure 1). H3K36me2 is mostly found in intergenic regions (Weinberg et al., 2019; Xu et al., 2020), while H3K36me3 localizes along gene bodies (Weinberg et al., 2019), and is proposed to serve as a hub to recruit proteins involved in RNA splicing (Pradeepa et al., 2012; Iwamori et al., 2016). Control of H3K36 methylation is modulated by H3K36 demethylases and methyltransferases. Mutations in these proteins have been associated with several neurodevelopmental disorders. Next, we highlight the cellular functions and neurological clinical findings associated with histone methyltransferases that deposit the H3K36me1/2/3 histone marks (NSD1, NSD2, ASH1L, SETD2, and SETD5) (Figure 1 and Tables 1, 2).

### 2.1. NSD1 gene dosage and the balancing act of large vs. small brains

Nuclear receptor SET domain-containing protein 1 (NSD1) catalyzes both the mono-methylation and di-methylation of H3K36 (Li et al., 2009). The fact that H3K36me1/2 could serve as a substrate for the tri-methylation of H3K36 (H3K36me3), suggests that NSD1 function might also contribute to the total levels of H3K36me3 (Lucio-Eterovic et al., 2010). Haploinsufficiency of *NSD1* has been associated with syndromic (Kurotaki et al., 2002; Buxbaum et al., 2007; Koshimizu et al., 2013; Lane et al., 2017) and idiopathic ASD (De Rubeis et al., 2014; Iossifov et al., 2015; Yuen et al., 2017; Satterstrom et al., 2020). Loss of function mutations in *NSD1* are a major cause of Sotos Syndrome (Kurotaki et al., 2002, 2005) which presents with macrocephaly, pre- and postnatal overgrowth, facial dysmorphism, ID, autistic features, developmental delay, and in some cases seizures (Sotos et al., 1964; Fortin et al., 2021). The macrocephaly phenotype associated with *NSD1* mutations in Sotos syndrome has variable penetrance as 72–95% of patients present with larger head circumference (Cecconi et al., 2005; Faravelli, 2005; Saugier-Veber et al., 2007; Fortin et al., 2021; Muhsin et al., 2022). In contrast, duplication of *NSD1* in the microduplication 5q35 syndrome leads to microcephaly in 74% of cases, with undergrowth, and developmental delay in close to 90% of the cases (Chen et al., 2006; Franco et al., 2010; Dikow et al., 2013; Rosenfeld et al., 2013; Quintero-Rivera et al., 2021). Therefore, differences in *NSD1* gene dosage that elicit gain or loss of function phenotypes result in either microcephaly or macrocephaly, respectively. However, the precise molecular and cellular mechanisms regulated by NSD1 that influence the control of brain size are underexplored.

At the molecular level, NSD1 is proposed to regulate the recruitment of RNA Polymerase II to bone morphogenetic protein 4 (BMP4) nascent mRNA, indicating a role for NSD1 in transcriptional activation during development (Lucio-Eterovic et al., 2010). In embryonic stem cells (ESCs), NSD1 counteracts PRC2 activity by preventing non-specific deposition of H3K27me3 along the genome, which will prevent random transcriptional gene repression by PRC2 (Streubel et al., 2018). Therefore, NSD1 promotes a chromatin environment that is permissive of transcriptional activation to ensure the normal progression of organismal development. In the developing human cortex, *NSD1* is highly expressed in RGCs, oRGCs, IPCs, and subsets of excitatory and inhibitory neurons suggesting a major role for *NSD1* in the regulation of these neural cell types (Speir et al., 2021). In fact, acute knockdown of *Nsd1* in mouse cortical progenitors led to migration defects in cortical neurons and correlated with differential expression of gene programs that regulate neuronal migration and cell fate decisions (Almuriekhi et al., 2015). In contrast, studies in *Nsd1* heterozygous mutant mice showed that while they did not present defects in overall brain architecture, they did show reduced social novelty which is a hallmark of an autism-like behavior (Oishi et al., 2020). There are currently no duplication mouse models for NSD1 function. However, overexpression studies in *Drosophila* showed that increased dosage of Nsd1 in glial cells lead to cell death of glial and neuronal cells resulting in smaller head sizes (Kim et al., 2020). Taken together these studies suggest that while NSD1 contributes to the development of the brain architecture and circuitry there might be cell type and species-specific differences associated with its function.

### 2.2. NSD2 influencing brain size through NPC proliferation and survival dynamics

Nuclear receptor SET domain-containing protein 2 (NSD2) is a catalyst for the di-methylation of H3K36. In humans, *NSD2* is located in a critical region of microdeletion 4p16.3 which is associated with Wolf-Hirschhorn syndrome (Bergemann et al., 2005), which presents with microcephaly at birth (Bernardini et al., 2018; Zanoni et al., 2021), global developmental delay, postnatal growth deficiency, ID, distinct craniofacial features, and seizures (Kagitani-Shimono et al., 2005; Battaglia et al., 2021; Wiel et al., 2022). Loss of function mutations in *NSD2* have been associated with ASD across multiple genetics studies (De Rubeis et al., 2014; Wang et al., 2016; Yuen et al., 2017), and replicate several of the clinical presentations of Wolf–Hirschhorn syndrome including microcephaly, global developmental delay, and ID, but do not present seizures (Boczek et al., 2018; Barrie et al., 2019; Wiel et al., 2022). Therefore, the clinical evidence suggests an essential role for *NSD2* in the development of human neuronal circuitry.

In non-neuronal cells, NSD2 is proposed to regulate cellular proliferation and survival (Park et al., 2018). Therefore, it is possible that the microcephaly phenotypes in humans with mutations in *NSD2* could arise from defects in NPC proliferation. Mouse models with different deletions that affect *Nsd2* present with a reduced cerebral cortex (Naf et al., 2001), while *Zebrafish* null for *nsd2* showed reduced numbers of motor neurons with delayed axon outgrowth (Yamada-Okabe et al., 2010; Yu et al., 2017). The reduction in cerebral cortex size in mice or the reduction in the numbers of motor neurons could be a consequence of reduced NPC proliferation, altered cell fate or increase neuronal cell death. However, additional mechanistic studies using human systems are needed to address the role of NSD2 in the control of brain size and neuronal function at large.

### 2.3. ASH1L a potential regulator of prenatal and postnatal brain growth

Similar, to the NSD proteins, the Absent, small, or homeotic-1 like (ASH1L) methyltransferase catalyzes both the mono- and di-methylation of H3K36 (H3K36me1, H3K36me2) (Tanaka et al., 2007, 2011). ASH1L might also be indirectly associated with tri-methylation of H3K4 (H3K4me3) (Gregory et al., 2007). *De novo* loss of function and missense mutations in *ASH1L* reached genome wide significance across large genetics studies in ASD (Willsey et al., 2013; Iossifov et al., 2014, 2015; Tammimies et al., 2015; Wang et al., 2016; Stessman et al., 2017). Mutations in *ASH1L* have also been reported in severe forms of ASD, presenting with ID, seizures, speech difficulties, and in a small number of cases microcephaly and macrocephaly (Homsy et al., 2015; Wang et al., 2016; Faundes et al., 2017, 2018; Okamoto et al., 2017; Stessman et al., 2017). Additional pathogenic variants in *ASH1L* have been associated with Attention-deficit/hyperactivity disorder (ADHD) (Satterstrom et al., 2019), Tourette syndrome (Liu et al., 2020; Zhang et al., 2021), and epilepsy (Tang et al., 2020). The association of *ASH1L* deficits with multiple neurological disorders suggests that common downstream mechanisms could underlie the diverse neuropathology in ASH1L-related disorders.

ASH1L is emerging as a major modulator of neurodevelopment and insight into its function has come from multiple studies that integrate mouse and human experimental systems. CRISPR-inactivation of *ASH1L* in LUHMES neural-like progenitor cells, which are of human mesencephalic origin and give rise to dopaminergic-like neurons, resulted in delayed neuronal differentiation (Lalli et al., 2020). Similarly, introduction of truncating frameshift mutations in *ASH1L* by CRISPR/CAS9 genome editing led to reduced neuronal output that correlated with increased neural stem cell production in a human induced pluripotent stem cell (iPSC)-model of prefrontal cortex neurogenesis (Cederquist et al., 2020). Together these studies suggest a role for ASH1L in the regulation of neuronal numbers. However, additional evidence suggests that ASH1L functions in a cell-type dependent manner affecting additional processes important for neuronal morphogenesis and synaptic function. For example, knockdown of *ASH1L* in human ESC-derived neurons led to severe reduction in neurite outgrowth (Lalli et al., 2020; Cheon et al., 2022). *In vivo*, knockout of *Ash1l* in mice lead to an overall growth retardation at later postnatal stages and revealed cortical malformations (Gao et al., 2021b). Specifically, knockout of *Ash1l* led to a disorganized cortical architecture, with SATB Homeobox 2 (*Satb2*), a marker specific for cortical layer 2/4 being observed in deeper layers (Gao et al., 2021b). These results suggested that *Ash1l* knockout led to the delayed lamination of neuronal cells. Recently, conditional knockout of *Ash1l* in mice demonstrated a role for ASH1L in synaptic function and synapse remodeling. *Ash1l* -haploinsufficiency resulted in neuronal hyperactivity (Gao et al., 2022) and increased synapse numbers due to deficits in synapse elimination (Yan et al., 2022). Together the clinical and mechanistic studies discussed above, suggest that ASH1L regulates gene programs relevant to the proper development of the brain architecture, wiring, and possibly the control of brain size.

### 2.4. SETD2 gain or loss of function differentially impact brain size

In contrast to the NSDs and ASH1L proteins, the Set Domain Containing 2 (SETD2) catalyzes exclusively the tri-methylation of H3K36 (Edmunds et al., 2008), which is a histone mark mostly found in the gene body of transcriptionally active genes. Several clinical studies suggest that loss of *SETD2* could impact neuronal connectivity and brain size. *De novo* loss of function mutations in *SETD2* have been associated with idiopathic ASD (O’Roak et al., 2012a,b; Wang et al., 2020), and a syndrome that presents with variable degrees of ID, autistic behaviors, overgrowth, seizures, speech delays, facial dysmorphisms, and macrocephaly (Lumish et al., 2015; van Rij et al., 2018; Marzin et al., 2019; Chen et al., 2021). In contrast, a recent report of patients with a gain of function mutation (p. Arg1740Trp) in *SETD2*, presented microcephaly and epilepsy in 12 out of 12 cases (Rabin et al., 2020). Therefore, SETD2 like the NSDs and ASH1L, appears to be important for the proper development of neuronal connectivity and for the control of brain size.

SETD2 is a multifaceted protein that regulates transcriptional activation, modulates transcriptional elongation through its interaction with RNA Polymerase II (Kizer et al., 2005), is essential in the control of co-transcriptional splicing (Leung et al., 2019), and contributes to DNA repair through promotion of homologous recombination (Pfister et al., 2014). In the human developing cortex *SETD2* is widely expressed in oRGCs, RGCs, IPCs, astrocytes, excitatory, and inhibitory neurons (Speir et al., 2021), which suggests widespread functions in multiple neural cell types. Hence cell specific knockout studies will be important to define cell-type specific mechanisms governed by SETD2 during the development of neuronal circuitry. In fact, knockout of *Setd2* in the developing forebrain revealed abnormal cortical arealization and aberrant thalamo-cortico-thalamic circuits in mice that also had defects in social interaction, motor learning and spatial memory (Xu et al., 2021). The observed neural phenotypes associated with SETD2 dysfunction could be related in part to changes in the chromatin environment. For instance, SETD2 interacts with histone H2 variant H2A.Z to regulate the H3K36me3 modification of the NK2 Homeobox 4 (Nkx2.4) gene (Shen et al., 2018; Zhang et al., 2020). Deficits in both Nkx2.4 and H2A.Z promote NPC proliferation and inhibit neuronal differentiation (Shen et al., 2018; Zhang et al., 2020). However, only deficits in H2A.Z lead to dendritic arborization defects (Shen et al., 2018). Together deficits in NPC proliferation, neuronal differentiation, and arborization could contribute to the defects in arealization and the aberrant thalamo-cortical circuits associated with loss of *Setd2* (Xu et al., 2021). Therefore, we posit that the modulation of the chromatin environment by SETD2 plays an important role in the regulation of cell fate transitions during neurogenesis, and the structural development of neuronal circuits.

### 2.5. SETD5 dysfunction in microcephaly related syndrome

Like SETD2, the SET Domain Containing 5 (SETD5) histone methyltransferase appears to exclusively catalyze the tri-methylation of H3K36 (Sessa et al., 2019). *SETD5* is one of 3 genes located in a critical region of the 3p25 microdeletion syndrome. Large deletions in 3p25 present with varying degrees of ID, and facial dysmorphisms and could include seizures, cardiac defects, and microcephaly, yet smaller deletions containing the critical interval only present with ID and facial dysmorphisms (Kellogg et al., 2013; Grozeva et al., 2014; Kuechler et al., 2015). Similarly, haploinsufficiency of *SETD5* has been identified as a frequent cause of ID based on whole exome sequencing studies (Kuechler et al., 2015) and has been associated with variable dysmorphic features, ASD, and ADHD (De Rubeis et al., 2014; Grozeva et al., 2014; Szczałuba et al., 2016; Rawlins et al., 2017; Fernandes et al., 2018; Powis et al., 2018; Satterstrom et al., 2020). Finally, loss of function mutation in SETD5 was identified in a patient with KBG syndrome that also presented with microcephaly (Crippa et al., 2020).

Loss of SETD5 causes abnormal transcription within neural stem cells (Nakagawa et al., 2020). In neonatal mouse brains, knockdown of *Setd5* led to an increase in the proliferation of cortical progenitor cells, and a significant decrease in glutamatergic synaptic formation (Sessa et al., 2019). In addition, *Setd5* haploinsufficiency in mice was associated with decreased neurite length, reduced arbor complexity and autism-like behaviors (Moore et al., 2019). Furthermore, a different study of mice haploinsufficient for SETD5 showed that despite an overall normal brain architecture and cortical lamination, the animals presented defects in ultrasonic vocalization and cognitive tasks (Deliu et al., 2018). Taken together these studies demonstrate an essential role for H3K36me3 methylation mediated by SETD5 during neurogenesis, neuronal differentiation, maturation, and synaptic function.

In summary, mounting evidence suggests a major role for H3K36 methyltransferases in the regulation of early brain development and the proper wiring of neural circuits. H3K36 methyltransferases have been implicated in multiple processes that can contribute to the control of brain size including cell fate transitions, NPC proliferation and neuronal arborization. Next, we present an overview of another group of histone Lysine methyltransferases with major roles in neuronal development.

## 3. H3K4 methylation and neurodevelopment

H3K4 methylation occurs in three states: mono-, di-, and tri-methylation (H3K4me, H3K4me2, and H3k4me3) and has previously been associated with transcriptional activation (Barski et al., 2007; Zhu et al., 2013; Figure 1). The three different H3K4 methylation states are differentially distributed across the genome, with H3K4me1 being present primarily at enhancer regions, while H3K4me2/3 are enriched at transcriptional active promoters (Collins et al., 2019). Histone H3K4 methyltransferases and demethylases control the transition between these different methylation states (Shilatifard, 2008). H3K4 histone methyltransferases have been widely studied in cancer biology (Deb et al., 2014; Li et al., 2018; Yang et al., 2021), and have been strongly implicated in various neurodevelopmental disorders, including ASD and ID (Wynder et al., 2010; Vallianatos and Iwase, 2015; Kim et al., 2017; Tables 3, 4).

### 3.1. MLL1/KMT2A a central contributor to the pathogenesis of multiple microcephaly associated syndromes

Mixed lineage-leukemia 1 (MLL1), also known as Lysine methyltransferase 2A (KMT2A), is a member of the Trithorax Group (TrxG) of proteins. MLL1/KMT2A catalyzes the mono, di and tri-methylation of H3K4 (H3K4me1/2/3). Dominant *de novo* mutations in *MLL1*/*KMT2A* are associated with Weidemann–Steiner syndrome. Patients with Weidemann–Steiner syndrome exhibit ID, microcephaly, short stature, and a subset present with ASD (Jones et al., 2012; Strom et al., 2014; Chan et al., 2019; Sheppard et al., 2021). Large scale exome sequencing studies identified mutations in *MLL1/KMT2A* among patients presenting with developmental delay and ID (Deciphering Developmental Disorders Study, 2015; Lelieveld et al., 2016; Trujillano et al., 2017). Clinical genetics studies identified *MLL1/KMT2A* pathogenic variants in patients diagnosed with either Kabuki syndrome or Rubinstein–Taiby syndrome which are characterized by ID, postnatal growth defects and microcephaly (Sobreira et al., 2017; Castiglioni et al., 2022).

MLL1/KMT2A-mediated H3K4 methylation has been associated with the regulation of transcriptional initiation through recruitment of RNA Polymerase II (Wang et al., 2009). Morpholino knockdown of the *MLL1/KMT2A* homologue in *Zebrafish* embryos showed reduced NPC proliferation, premature neuronal differentiation, and reduced numbers of glial cells (Huang et al., 2015). Similarly, in human iPSC-derived neural stem cells loss of function mutations in *MLL1/KMT2A* introduced by CRISPR/CAS9 genome editing led to neurogenesis defects that resulted in the depletion of the neural stem cell population and premature neuronal differentiation (Cederquist et al., 2020). In contrast, *Mll1/Kmt2a*—deficient SVZ adult neural stem cells showed severe impairment of neuronal differentiation but normal gliogenesis (Lim et al., 2009). In addition to its role in neurogenesis, MLL1/KMT2A also modulates synaptic function. Mice heterozygous for *Mll1/Kmt2a* showed reduced numbers of dendritic spines (Vallianatos et al., 2020), and the specific ablation of *Mll1/Kmt2a* in mice prefrontal cortical neurons lead to defects in short-term synaptic plasticity (Jakovcevski et al., 2015). Furthermore, the synaptic defects identified in *Mll1/Kmt2a* mutant mice lead to increased aggressive behaviors and social dominance (Vallianatos et al., 2020), as well as deficits in working memory (Jakovcevski et al., 2015). In summary, MLL1/KMT2A control of H3K4 methylation might be species and cell type specific but is overall essential for the proper development of brain architecture and wiring.

### 3.2. MLL2/KMT2B in dystonia and brain size control

Mixed lineage-leukemia 2 (MLL2), also known as Lysine methyltransferase 2B (KMT2B), tri-methylates H3K4 (Denissov et al., 2014). Mutations in *MLL2/KMT2B* have been commonly associated with early onset generalized dystonia, which is a disorder characterized by sustained muscle contractions causing abnormal, repetitive movements, and in a small number of cases has been associated with ID, facial dysmorphisms and microcephaly (Albanese et al., 2013; Zech et al., 2016; Meyer et al., 2017; Kawarai et al., 2018). In contrast, a subset of non-dystonia patients with mutations in *MLL2/KMT2B* presented with neurodevelopmental delay, ID, microcephaly, short stature, and facial dysmorphic features (Cif et al., 2020).

Knockout of *Mll2/Kmt2b* in mouse ESCs resulted in a severe delay in differentiation toward the ectodermal lineage which gives rise to the neuroectoderm (Lubitz et al., 2007). Further, inactivation of *Mll2/Kmt2b* resulted in impaired trans differentiation of mouse fibroblasts into neuronal cells, and disruption of a network of genes responsible for neuronal maturation, suggesting that MLL2/KMT2B is required for the activation of the neuronal maturation programs during this process (Barbagiovanni et al., 2018). *In vivo*, post-natal deletion of *Mll2/Kmt2b* from excitatory forebrain neurons led to impaired memory consolidation (Kerimoglu et al., 2013). Therefore, similar to MLL1/KMT2A, MLL2/KMT2B is also essential for the regulation of gene networks that modulate memory processes. However, the gene programs modulated by both proteins are almost non-overlapping (Kerimoglu et al., 2017).

### 3.3. MLL3/KMT2C

Mixed lineage-leukemia 3 (MLL3), also known as Lysine methyltransferase 2C (KMT2C), catalyzes the mono- and di-methylation of H3K4 (Hu et al., 2013; Lee et al., 2013). H3K4me and H3K4me2 have been identified at enhancer regions leading to transcriptional activation (Hu et al., 2013). Pathogenic mutations in *MLL3* are associated with Kleefstra syndrome, ID, behavioral problems, and epileptic seizures as its core features, while in some cases microcephaly is present (Kleefstra et al., 2012; Frega et al., 2020). Pathogenic variants in *MLL3* were identified in large whole exon sequencing studies in patients with ASD (De Rubeis et al., 2014; Iossifov et al., 2014), and have been associated with an ID/ASD related syndrome that presents microcephaly in 50% of the cases reported (Koemans et al., 2017). Therefore, suggesting the importance of this member of the MLL family of proteins in the development of neuronal circuitry.

*MLL3/KMT2C* is widely expressed in the developing human fetal brain (Johnson et al., 2009). Introduction of frameshift mutations in *MLL3/KMT2C* by CRISPR/CAS9 genome editing in iPSC-derived neural stem cells render them unable to generate neurons (Cederquist et al., 2020). Work in other species suggests that *MLL3/KMT2C* might have additional roles outside of the control of neurogenesis, as knockdown of *MLL3/KMT2C* in rat primary neurons led to a hyperactive state of mature neuronal networks compared to control neurons (Frega et al., 2020). In *Drosophila*, knockdown of the *MLL3/KMT2C* homologue in the adult nervous system led to reduced short-term memory, but no effect on cell fate decisions or increase in cell death was observed (Koemans et al., 2017). Taken together, these studies across multiple species suggest that *MLL3/KMT2C* drives temporally distinct programs as development proceeds.

### 3.4. MLL4/KMT2D in Kabuki syndrome associated with microcephaly

Like MLL3/KMT2C the paralogous MLL4/KMT2D can also catalyze the mono- and di-methylation of H3K4 (Hu et al., 2013; Lee et al., 2013). Mutations in *MLL4/KMT2D* have been identified as the most common cause of Kabuki syndrome, which is characterized by distinctive facial features, microcephaly, growth delay, cardiac anomalies, and varying degrees of ID (Lehman et al., 2017; Di Candia et al., 2022; Usluer et al., 2022; Table 3).

Work in human iPSC-derived NPCs from a Kabuki syndrome patient with a nonsense mutation in *MLL4/KMT2D* showed a reduction in NPC proliferation that resulted in precocious neuronal differentiation (Carosso et al., 2019). Similarly, a study of a mouse with a truncation mutation in the catalytic domain of *Mll4/Kmt2d* led to reduced neurogenesis of the granule cell layer. Taken together, these studies suggest a major role for MLL4/KMT2D regulating the numbers of NPCs during embryonic and adult neurogenesis.

### 3.5. SETD1A is a major factor in neuropsychiatric disorders

SET Domain Containing 1A (SETD1A) catalyzes the tri-methylation of H3K4 (Lee and Skalnik, 2008), and has been shown to be important for the maintenance of genome stability during DNA replication (Higgs et al., 2018). Haploinsufficiency of *SETD1A* was identified in patients with a novel neurodevelopmental disorder, characterized by global developmental delay, ID, facial dysmorphisms, psychosis, behavioral and psychiatric abnormalities, and in some cases autistic-like behaviors (Kummeling et al., 2021). In contrast, a *de novo* mutation in *SETD1A* was associated with macrocephaly but was not co-diagnosed with ASD (Zhang et al., 2022). Recently, a large human genetics study of schizophrenia identified rare coding variants in *SETD1A* raising above genome-wide significance (Singh et al., 2022). Clearly, *SETD1A* has a major role in the proper development of neuronal circuits, however, additional evidence is needed to determine how common is the macrocephaly phenotype in patients with SETD1A mutations.

SETD1A has been proposed to regulate neuronal progenitor proliferation through its interaction with Histone Cell Cycle Regulator (HIRA) (Li and Jiao, 2017) could contribute to the regulation of neuronal differentiation and excitability by transcriptionally activating β-catenin (Li and Jiao, 2017), a key component of the Wnt/β-catenin pathway which is essential for neurogenesis (Hirabayashi et al., 2004; Zhang et al., 2011; Rubio et al., 2020). In mice, a missense mutation in SETD1A resulted in faster migration of neurons within the cortex (Yu X. et al., 2019), suggesting that neuronal migration could also be regulated by SETD1A. Mice heterozygous for *Setd1a* showed decreased axonal branching, reduced dendritic spines, and altered cortical synaptic dynamics that are associated with defects in working memory and social interaction (Mukai et al., 2019; Nagahama et al., 2020). In contrast, iPSC-derived human glutamatergic and GABAergic neurons haploinsufficient for *SETD1A* had enlarged neuronal arbors, increased network activity, and synaptic connectivity (Wang et al., 2022). Taken together, mounting evidence suggests an essential role for SETD1A in the regulation of neurogenesis and neuronal connectivity, that might differ in a species-specific manner.

### 3.6. SETD1B and the balancing act between a large vs. a small brain

SET Domain Containing 1B (SETD1B) is the catalytic subunit of the Complex Proteins Associated with SET1 (COMPASS) that catalyzes the tri-methylation of H3K4 (Lee et al., 2007). Human pathogenic variants in coding regions of *SETD1B* have been associated with ASD, ID, and epilepsy (Den et al., 2019; Hiraide et al., 2019; Roston et al., 2021). Additionally, *SETD1B* has been identified as a candidate gene for 12q24.31 microdeletion syndrome, which presents with ID, autism-like features, facial dysmorphisms, and epilepsy (Baple et al., 2010; Palumbo et al., 2015; Labonne et al., 2016). Both microcephaly and macrocephaly have been associated with SETD1B mutations. However, this is a variable phenotype as in each only 2 out of 36 patients were reported to have significant changes in head circumference (Weerts et al., 2021). In mice, deletion of *Setd1b* at early embryonic stages did not appear to be required for neural stem cell (NSC) survival or proliferation (Bledau et al., 2014). However, postnatal deletion of *Setd1b* in excitatory forebrain cortical neurons led to severe learning impairment and altered the expression of neural genes involved in learning and memory (Michurina et al., 2022). Therefore, SETD1B appears to control primarily neuronal function.

In summary, the different H3K4 methyltransferases control various stages of neuronal development that can influence brain size and cognition by controlling different H3 methylation states that influence distinct gene programs.

## 4. The role of H3K27 methylation by PRC2 in neurodevelopment and brain size control

Polycomb group complex genes are a family of chromatin regulators closely associated with gene silencing. In particular, PRC2 plays a vital role in the epigenetic regulation of gene expression during normal development (Deevy and Bracken, 2019). PRC2 catalyzes H3K27me3 (Yu J. et al., 2019), which is a repressive histone modification associated with gene silencing (Figure 1). PRC2 is composed of four core subunits: EZH1/2, SUZ12, EED, and RbAp46/48. Enhancer of Zeste Homolog 1 or 2 (EZH1/2) is the main catalytic subunit of PRC2 and it catalyzes the mono-, di-, or tri-methylation of H3K27 (Di Croce and Helin, 2013). Embryonic ectoderm development (EED) subunit of PRC2 plays an important role in maintaining H3K27me3 through the binding of H3K27me3 to its C-terminal domain. Suppressor of zeste 12 (SUZ12) helps to maintain the stability and catalytic activity of EZH2. Retinoblastoma-associated protein 46/48 (RbAP46/48) is required for the association of PRC2 to histone tails.

Different core subunits of PRC2 have been linked to multiple neurodevelopmental disorders. Mutations in EZH2 (catalytic subunit of PRC2) have been associated with Weaver Syndrome, which is characterized by overgrowth and ID (Tatton-Brown et al., 2013). Further, mutations in EZH2 have also been suggested to contribute to the genetic ethology of ASD in the Chinese Han population (Li et al., 2016). Previous studies suggest that a subset of ASD cases might be associated with larger brain size (Sacco et al., 2015). However, the extent to which ASD patients with EZH2 pathogenic variants also present macrocephaly is unknown. Similar to EZH2, several mutations in EED have also been identified in patients with Weaver syndrome (Cooney et al., 2017; Cyrus et al., 2019; Griffiths et al., 2019), while mutations in SUZ12 have been associated with a Weaver-like syndrome presenting with macrocephaly, postnatal overgrowth, and skeletal abnormalities (Imagawa et al., 2017, 2018). Similarly, mutations in non-core subunits of PRC2 have also been shown to have an important role in neuronal development. Jumonji and AT-Rich Interaction Domain Containing 2 (JARID2) is a non-core subunit of PRC2 that acts as an activator of Polycomb (Kasinath et al., 2021). Mutations in JARID2 have been associated with developmental delay, variable degrees of ID, facial dysmorphisms (Verberne et al., 2021), and ASD (Weiss et al., 2009; Ramos et al., 2012). However, to date no brain size alterations correlate with pathogenic variants in JARID2.

Based on the clinical studies, PRC2 has an important role in neuronal development and can impact brain size. We posit that PRC2 modulates brain growth through the control of gene programs that impact neural cell fate decisions and neuronal morphogenesis (Pereira et al., 2010; Liu et al., 2018). In particular the tight regulation of the switch from a neurogenic to an astrocytic cell fate could alter brain size. Knockdown of EED in mice NPCs showed a lengthening of the neurogenic phase while there was a shortening of the astrocytic phase (Hirabayashi et al., 2009). Similarly, deletion of EZH2 in cortical progenitors led to an increase in the number of neurons (Pereira et al., 2010) which correlated with a lengthening of the neurogenic phase (Hirabayashi et al., 2009). Loss of EZH2 in primary neurons led to increased neuronal arborization and advanced migration of cells to the upper layers (Pereira et al., 2010). Alterations in neuronal arborization is a mechanism that could contribute to changes in brain size postnatally. The defects in neuronal arborization associated with EZH2 dysfunction might be mediated in part by alterations in the Brain-derived Neurotrophic Factor (BDNF)/TrkB signaling pathway which is a major regulator of neuronal arborization, synaptic function and survival (Gonzalez et al., 2016). EZH2 regulates BDNF expression through its interaction with Chromodomain Y Like (CDYL). CDYL recognizes H3K27me3 and recruits additional PRC2, leading to a restriction of dendritic morphogenesis (Qi et al., 2014), which could result from increased repression of gene expression by elevated levels of H3K27me2 repressive marks. Similarly, deletion of Lysine demethylase 6A (UTX), an H3K27 demethylase, resulted in abnormal dendritic development and reduced synaptic formation coinciding with increased levels of H3K27me3 (Tang et al., 2017). Taken together, these results implicate the restriction of PRC2 activity as essential for proper neuronal differentiation, migration, and morphogenesis.

## 5. Counteracting activities between H3K36 and H3K4 methylation and PRC2-mediated H3K27 methylation

The interplay between PRC2 methylation of H3K27 and Trithorax methylation of H3K36 in the regulation of gene expression is a known mechanism essential during development. However, the implications of PRC2/Trithorax counteracting activities in the formation of proper neuronal architecture and circuitry are largely understudied (Schuettengruber et al., 2017). Deposition of H3K36 methylation marks provides a steric hindrance to PRC2 activity by preventing it from methylating H3K27me3. The majority of our current knowledge on how these counteracting epigenetic mechanisms work is based on studies of non-neuronal systems. For example, knockdown of *Nsd1* in mouse ESCs resulted in a genome-wide reduction of H3K36me2 levels coupled with increased levels in H3K27me3. Additionally, NSD1 is required in ESCs for the demarcation of H3K27me3 and H3K27me2, further corroborating the importance of the counteracting activity between H3K36 histone methyltransferase activity and PRC2 activity (Streubel et al., 2018). However, the mechanism by which NSD1 regulates the demarcation of H3K27me3 and H3K27me2 in neurons is not currently known. RNA-sequencing of a *Nsd1* knockout mouse revealed counteracting regulation of PRC2-associated genes (Shirane et al., 2020). Knockdown of both *Nsd1* and *Nsd2* in mice brown preadipocytes resulted in decreased H3K36me2 and increased H3K27me3, with a more substantial change seen when *Nsd2* was knocked down compared to *Nsd1* (Zhuang et al., 2018). Similarly, in *Drosophila*, ASH1L antagonizes PRC2 activity by preventing the methylation of H3K27me3 (Yuan et al., 2011). *ASH1L* knockdown in bovine cumulus cells resulted in increased mRNA expression of *EZH2* and *SUZ12* which in turn could lead to higher levels of H3K27me3 methylation. These studies demonstrate that the opposing activities of H3K36 modifying enzymes and PRC2-mediated methylation of H3K27 is a conserved epigenetic mechanism. ASH1L has also been shown to form a functional interaction with CREB-binding protein (CBP) in *Drosophila* (Bantignies et al., 2000). CBP is a histone acetyltransferase that acetylates H3K27, preventing the methylation of H3K27 (Tie et al., 2014). Therefore, ASH1L by directly methylating H3K36me2 and indirectly through its interaction with CBP could oppose the catalytic activity of PRC2.

However, despite the antagonizing relationship between H3K36 methyltransferase activity and PRC2 activity, there is some evidence that PRC2 directly binds to regions containing H3K36 methylation. The introduction of PRC2 to H3K36 methylated regions is driven by PHD Finger Protein 1 (PHF1) and PHD Finger Protein 19 (PHF19) (Cai et al., 2013), which contain Tudor domains that recognize H3K36me3. Recognition of H3K36 methylation marks by PHF1 or PHF19 promotes the introduction of PRC2, leading to gene silencing in these regions. PHF1 is also important for the stabilization of PRC2 on chromatin (Cai et al., 2013). These results suggest a more direct interaction between these two histone methylation marks.

Finally, besides the opposing activities of H3K36 vs. H3K27 methylation states, H3K4 methylation also provides a counterbalancing mechanism to H3K27 modifiers. Mechanistically, H3K4 histone marks are proposed to indirectly prevent the methylation of H3K27 (Tie et al., 2014). Specifically, methylated H3K4 binds CBP, which by acetylating H3K27 prevents its methylation (Tie et al., 2014). Comparably, H3K27 tri-methylation has been shown to inhibit the binding of the SET1-like H3K4 methyltransferase complex to histone H3 (Kim et al., 2013). In addition, the MLL3/4 COMPASS-like complex contains the histone demethylase KDM6/UTX, which is able to remove the H3K27me3 repressive mark produced by PRC2 (Agger et al., 2007; Kim et al., 2014; Piunti and Shilatifard, 2016). Therefore, there is an interplay of opposing activities between H3K4 and H3K27 modifying enzymes that modulate the transition between transcriptional activation and repression (Piunti and Shilatifard, 2016; Schuettengruber et al., 2017).

## 6. Impact of histone methyltransferases on brain size and circuitry development–Integrating the clinical findings with the underlying biological mechanisms

### 6.1. Microcephaly

Different cellular mechanisms can contribute to a reduced brain size. Here we discuss two major forms of microcephaly (primary and postnatal) associated with genetic causes and the cellular mechanisms that could underlie the development of a microcephalic brain. Primary microcephaly is characterized by a small brain size in the absence of major brain malformations at birth. Some of the major cellular mechanisms that underlie primary microcephaly pathogenesis are reduced NPC proliferation, increased cell death of NPC, and changes in cell fate determination (associated with premature cell cycle exit) (Wollnik, 2010). This is not surprising as several of the key primary microcephaly genes (CDK5RAP2, ASPM, WDR62, and MCPH1) (Jackson et al., 2002; Bond et al., 2005; Shen et al., 2005; Yu et al., 2010) are essential for the control of mitosis and cell cycle progression (Cox et al., 2006; Jean et al., 2020).

Alternately, postnatal microcephaly has been associated with defects in axon and dendritic arborization (Kwon et al., 2006; Dindot et al., 2008; Belichenko et al., 2009; Zikopoulos and Barbas, 2010), synaptogenesis (Giedd et al., 1999), gliogenesis (Zuchero and Barres, 2015) and potentially increased postnatal neural cell death. In particular, defects in the BDNF/TrkB signaling pathway might constitute a common molecular underpinning of neurodevelopmental disorders associated with postnatal microcephaly. Variants in NTRK2, the gene encoding the BDNF receptor TrkB, have been identified in patients presenting with microcephaly (Yoganathan et al., 2021). During neuronal development the BDNF/TrkB signaling pathway is a major regulator of axonal and dendritic arborization (Gonzalez et al., 2016), as well as synaptogenesis (Yoshii and Constantine-Paton, 2010). Christianson syndrome (Ouyang et al., 2013) and Angelman syndrome (Judson et al., 2017) are two neurodevelopmental disorders associated with postnatal microcephaly that share defects in neurotrophin signaling. Mouse models of both syndromes showed reductions in neuronal arborization that correlate with deficits in neurotrophin signaling. However, the extent to which the different histone methyltransferases that have been associated with postnatal microcephaly could modulate brain size by modulating the BDNF/TrkB signaling pathway is underexplored. Recently, mice with loss of *Ash1l* revealed growth delays and ASD/ID-like social behaviors (Gao et al., 2021b). Knockdown of *ASH1L* in human ESC-derived cortical neurons lead to decreased neurite length and arbor complexity (Cheon et al., 2022). Similarly, depletion of ASH1L in human iPSC-derived NPCs resulted in delayed neural differentiation and maturation (Lalli et al., 2020). The neuronal arborization defects associated with loss of ASH1L might correlate with defects in the BDNF/TrkB signaling pathway (Cheon et al., 2022). Taken together, these data are suggestive of a mechanism through which deficits in ASH1L could underlie postnatal microcephaly through its regulation of the neurotrophin signaling pathway.

In addition to the potential role of ASH1L in the control of brain size postnatally, clinical studies suggest that mutations in either *NSD1, NSD2, SETD2*, or *SETD5* have also been implicated in microcephaly. Several clinical studies on NSD1 suggest that the microcephaly associated with *NSD1* increased dosage appears to be postnatal (Dikow et al., 2013). Based on overexpression studies in *Drosophila*, elevating the levels of the *NSD1* fly homologue in glial cells but not in neuronal cells led to reduced brain size that was associated with increased cell death of both glial and neuronal cells (Kim et al., 2020). However, further work in animal models and human iPSC-derived neurons with duplication of *NSD1* will be necessary to elucidate the role of this histone methyltransferase in the control of brain size in vertebrates. In contrast to patients with *NSD1* duplications, patients with small 4p.16.3 deletions that implicate *NSD2* have been reported to be microcephalic at birth (Bernardini et al., 2018) which suggests that NSD2 controls brain size by mechanisms independent of NSD1. Mouse models with different deletions that affect *Nsd2* present with reduced cerebral cortex, and reduction of *Nsd2* in zebrafish led to reduced numbers of motor neurons; however, to date there is a lack of mechanistic studies in these systems (Naf et al., 2001). In hematopoietic B cells, loss of NSD2 reduced B cell proliferation and led to alterations in cell fate commitment (Campos-Sanchez et al., 2017). Therefore, it is possible that during brain development, NSD2 could control the proliferation and cell fate decisions of NPCs which if impaired could be a mechanism contributing to the congenital microcephaly associated with deficits in NSD2.

In contrast, multiple mechanisms could be implicated in microcephaly associated with deficits in SETD5. For example, decreased formation of glutamatergic synapses was observed in mice with knockdown of *Setd5* (Sessa et al., 2019), which suggests defects in synaptogenesis that could lead to a reduced brain size postnatally. Similarly, defects in neuronal arborization outgrowth and complexity due to *Setd5* downregulation could also be a potential mechanism contributing to SETD5-associated microcephaly (Moore et al., 2019). However, based on the current clinical literature it is not clear whether the microcephaly associated with SETD5 is congenital or postnatal.

Additional insights into the mechanistic underpinnings of microcephaly associated with histone methyltransferases will be gained by the study of the MLL family of H3K4 methyltransferases. Deficits in both MLL1 and MLL2 have been associated with delays or impairments of neuronal differentiation observed in mice models targeting these two genes (Lubitz et al., 2007; Lim et al., 2009). Similar to MLL1, loss of MLL4 leads to reduced NPC proliferation (Lim et al., 2009; Huang et al., 2015; Carosso et al., 2019). Therefore, convergent downstream mechanisms modulated by MLL1, MLL2, and MLL4 could lead to a reduced brain size. In contrast, knockdown of MLL3 in rat cortical neurons showed increased hyperactive neuronal networks which correlated with decreased inhibitory and excitatory synaptic puncta (Frega et al., 2020). However, MLL3 is widely expressed in developing human fetal brain which suggests that it could have additional important functions during human neuronal development (Nagase et al., 2000; Johnson et al., 2009). To gain further understanding of the chromatin regulatory mechanisms that control brain size, studies that consider the counterbalance between MLLs’ driven H3K4 methylation and PRC2 driven H3K27 methylation will be needed. PRC2 is known to repress genes implicated in neural differentiation (Dietrich et al., 2012). Therefore, genome wide scale loss of H3K4 methylation could result in increased levels of H3K27me3 which could reduce neuronal differentiation for example.

### 6.2. Macrocephaly

Macrocephaly or enlarged brain in the absence of hydrocephalus has been linked to defects in axon/dendritic arborization, synaptogenesis and hyperproliferation of neuronal progenitors. A classic molecular mechanism previously implicated in macrocephaly is the deletion of Phosphatase and Tensin Homolog (PTEN). Loss of PTEN in neuronal cell populations of the mouse cerebral cortex resulted in progressive macrocephaly, axonal hypertrophy, and increased synapse formation (Kwon et al., 2006). PTEN is involved with the PI3K/AKT pathway, which regulates neurite growth and dendritic arborization (Crowder and Freeman, 1998; Markus et al., 2002; Jaworski et al., 2005). Mutations in Mammalian target of rapamycin (MTOR), which is downstream of the PI3K/AKT pathway, have been identified in patients with macrocephaly (Baynam et al., 2015). The MTOR pathway has been identified as important for proper synaptogenesis, suggesting a mechanistic role for abnormal synaptogenesis in the etiology of macrocephaly (Takei et al., 2004).

The mechanism through which loss of function mutations in *NSD1* contribute to macrocephaly in patients with Sotos Syndrome and Weaver Syndrome is largely understudied. Mice heterozygous for *Nsd1* presented deficits in social behaviors but did not show enlarged brain size (Oishi et al., 2020). Analysis of homozygous *Nsd1* mutant embryos at embryonic day 9.5 showed they had a smaller prosencephalon which was unexpected based on the human clinical findings on Sotos syndrome (Oishi et al., 2020). Therefore, there could be species specific differences, and it will be important to model Sotos syndrome using human organoids for example. Macrocephaly has been shown to be co-morbid with cortical malformations that could result from impaired neuronal migration (Andrews and Hulette, 1993; Robertson et al., 2000; Tenney et al., 2011; Mirzaa and Poduri, 2014; Winden et al., 2015). In humans, mutations in Adenomatous Polyposis coli 2 (APC2), a protein with a crucial role in the control of neuronal migration and axon guidance (Mimori-Kiyosue et al., 2000; Shintani et al., 2009, 2012), have been identified by whole exome sequencing as an additional cause of Sotos syndrome (Almuriekhi et al., 2015). APC2 is a downstream target of NSD1 as overexpression of *Apc2* in mice with *Nsd1* knockdown rescued the neuronal migration phenotype associated with loss of *Nsd1* (Almuriekhi et al., 2015). In contrast to the *Nsd1* mutant mice that does not show an enlarged head circumference, loss of function mutation in *Apc2* resulted in a Sotos Syndrome-like phenotype in mice, with increased head circumference and dilated brain ventricles (Almuriekhi et al., 2015). Similar to NSD1, both SETD2 and SETD1A have also been implicated in the control of neuronal migration (Yu X. et al., 2019; Xu et al., 2021). However, divergent mechanisms could underlie a larger head size associated with loss of function mutations in *SETD2* and *SETD1A*. For instance, deficits in SETD2 could increase NPC proliferation and lead to an enlarged brain (Li and Jiao, 2017; Shen et al., 2018; Xu et al., 2021). In contrast, loss of SETD1A might result in a macrocephalic brain due to increased neuronal arborization (Wang et al., 2022).

Impairing the function of EZH2, the catalytic subunit of PRC2, leads to increased neuronal differentiation which correlates with decreased NPC proliferation and accelerated gliogenesis (Liu et al., 2017). While it is unclear whether these cellular mechanisms contribute to PRC2-associated macrocephaly, as brain size was not investigated in this EZH2-knockout mouse model, an additional mechanism that could contribute to the increase in brain size associated with PRC2 defects is increased neuronal arborization (Di Meglio et al., 2013; Qi et al., 2014). Studies on the effects of PRC2 dysregulation and its interaction with CDYL provide support to the idea of increased neuronal arborization as a mechanism underlying PRC2-associated macrocephaly. The interaction between CDYL and EZH2 leads to inhibition of BDNF (Qi et al., 2014). If CDYL is unable to recruit EZH2 to inhibit BDNF, this could potentially lead to increased neuronal arborization and possibly synaptogenesis through upregulation of the BDNF/TrkB pathway. In fact, loss of EZH2 has been associated with increases in neuronal arborization and astrogliogenesis (Hirabayashi et al., 2009; Corley and Kroll, 2015), providing additional mechanisms that could contribute to PRC2-associated macrocephaly.

## 7. Final remarks

Brain size abnormalities, including microcephaly and macrocephaly, have previously been identified in a variety of neurodevelopmental disorders. Gaining insight into the mechanisms that produce brain size abnormalities will increase our understanding of the pathogenesis resulting in these neurodevelopmental disorders. Mutations in H3K36 and H3K4 histone methyltransferases have been associated with both microcephaly and macrocephaly related disorders (Figure 2 and Tables 1, 3). Methylation of H3K36 is vital to a variety of cellular processes, including transcriptional activation, DNA repair, and RNA splicing (Wang et al., 2007; Pradeepa et al., 2012; Huang and Zhu, 2018). Similarly, H3K4 methylation is also important to many cellular processes, including transcriptional activation and DNA repair (Barski et al., 2007; Zhu et al., 2013). Studies have shown a major role for H3K36 and H3K4 methylation during neuronal development. Histone methyltransferases responsible for H3K36 and H3K4 methylation have been implicated in neural stem cell proliferation (Zhang et al., 2011; Sessa et al., 2019), neuronal migration (Almuriekhi et al., 2015; Yu X. et al., 2019), neuronal arborization (Moore et al., 2019; Lalli et al., 2020; Gao et al., 2021b), and the control of gliogenesis which constitute essential mechanisms for proper brain development.

Additionally, gene silencing *via* PRC2-mediated H3K27 methylation is implicated in many different neurodevelopmental processes, including neuronal migration (Pereira et al., 2010), neural stem cell proliferation, and neuronal differentiation (Pereira et al., 2010; Dietrich et al., 2012). Mutations within the subunits of PRC2 are associated with several neurodevelopmental disorders, some of which also have reported brain size abnormalities (Cooney et al., 2017; Imagawa et al., 2017, 2018; Cyrus et al., 2019; Griffiths et al., 2019; Verberne et al., 2021). Both H3K36 and H3K4 methylation can directly or indirectly counteract PRC2 function by preventing the methylation of H3K27me3 (Agger et al., 2007; Yuan et al., 2011; Kim et al., 2014; Piunti and Shilatifard, 2016; Schuettengruber et al., 2017; Streubel et al., 2018; Zhuang et al., 2018; Shirane et al., 2020). While the balance between H3K36 and H3K4 methyltransferases and PRC2-mediated H3K27 tri-methylation have been well-studied in cancer and general development, the potential role of this counteracting activity to the etiology of different neurodevelopmental disorders is understudied. Potential increases in H3K27me3 caused by mutations in H3K36 or H3K4 methyltransferases could provide a convergent epigenetic mechanism leading to abnormal neurodevelopment.

It is important to note that the activity of proteins associated with H3K36 and H3K4 methylation is not necessarily restricted to only histone methylation. There is increasing evidence that several H3K36 methyltransferases also function as methylators of cytoskeletal components. Specifically, recent studies have shown that SETD2 is responsible for the trimethylation of Lysine 40 on α-tubulin (Park et al., 2016; Koenning et al., 2021). This cytoskeletal methylation has been identified in the mouse brain, with high concentrations of this mark found in post-mitotic neurites and growth cones (Koenning et al., 2021). Mutations to the Set2-Rpb1 interaction (SRI) domain of SETD2, which is responsible for substrate recognition, resulted in increased anxiety-like behavior and reduced dendritic arborization and axon length in mice (Koenning et al., 2021). Both α- and β-tubulin have been associated with maintenance of brain size, as mutations in both of these genes have been associated with microcephaly (Keays et al., 2007; Breuss et al., 2012). Additionally, SETD2 has been shown to tri-methylate actin on Lysine 68 through its interaction with the Huntington protein and the actin-binding adapter HIP1R (Seervai et al., 2020). This interaction was shown to be important for proper cell migration in human embryonic kidney 293T cells (Seervai et al., 2020). However, it is unknown if this interaction contributes to neuronal cell migration. SMYD2 is another H3K36 methyltransferase that has been linked to methylation of α-tubulin at Lysine 394 through cross-talk with cyclin-dependent kinases 4 and 6 (CDK4/6) (Li et al., 2020). Taken together, these studies suggest that direct methylation of cytoskeletal components is another mechanism that should be considered when attempting to uncover epigenetic mechanisms contributing to brain size abnormalities.

Epigenetic mechanisms play an important role in the regulation of gene programs responsible for brain development (Keverne, 2014). Here we have discussed how mutations across different histone methyltransferases lead to dysregulation of neurodevelopmental process, including NPC proliferation, neuronal migration, and neuronal arborization. Additionally, we connect these neurodevelopmental processes to brain size abnormalities identified in disorders associated with multiple histone methyltransferases. The counteracting mechanisms associated with H3K27me3 vs. H3K36me2 or H3k4me3 modifications bring forward the exciting opportunity to test rescue strategies of the different neural phenotypes. For example, inhibition of PRC2 catalytic activity using a pre-clinical inhibitor of EZH2 rescued ASH1L neuronal arborization defects in human ESC-derived neurons (Cheon et al., 2022). Similarly, acetylation of H3K27, which can inhibit the methylation of the same site by PRC2, has been used as a rescue strategy for H3K4- and H3K36-related neuronal phenotypes. For instance, inhibition of histone deacetylation which would maintain chromatin in a hyperacetylated state improved autistic-like behaviors of an *Ash1l* mutant mice (Gao et al., 2021a). Therefore, gaining an in depth understanding of the molecular signatures and cellular phenotypes associated with the pathogenic mutations in H3K36 and H3K4 histone methyltransferases in the etiology of various neurodevelopmental disorders will be instrumental in the potential development of mechanistic based therapeutic strategies.

Finally, a major remaining question moving forward in the field of neurodevelopmental epigenetics will be how to explain the phenotypic divergency associated with mutations in the different H3K4-H3K36- and H3K27 methyltransferases. Are the differences associated with cell type specific expression? Based on single cell data from developing human cortex *ASH1L, KMT2A, KMT2C, SETD2*, and *SETD5* are widely expressed in multiple subtypes of NPCs, inhibitory and excitatory neurons as well as glial cells (Nowakowski et al., 2017; Speir et al., 2021). In contrast, *NSD1, KMT2B, KMT2D, SETD1A*, and *SETD1B* seem to have higher expression in different NPC subtypes but are not as highly expressed in excitatory and inhibitory neurons in the developing cortex (Nowakowski et al., 2017; Speir et al., 2021). Therefore, the differences in spatial and temporal expression could contribute to the divergence of phenotypes associated with mutations in the different histone methyltransferases. In addition, despite having similar cellular functions the differences in the gene programs regulated by the different histone methyltransferases could also contribute to the phenotypic diversity associated with disruptions in the different enzymes. For example, the paralogous enzymes *MLL1/KMT2A* and *MLL2/KMT2B* contribute to memory formation through the regulation of distinct gene programs (Kerimoglu et al., 2017). The differences in substrate specificity and fine control of the chromatin environment, could be further contributed by the different complexes these enzymes form part of, which can give them their target specificity (Piunti and Shilatifard, 2016). Therefore, future studies will need to address the dynamic nature of these interactions during cortical development.

## Author contributions

Both authors designed, illustrated, wrote, and edited the manuscript and approved the submitted version.
